# The Effect of Rice Bran Extract on Arterial Blood Pressure, Hepatic Steatosis, and Inflammation in Mice Fed with a High-Fat Diet

**DOI:** 10.1155/2020/8374287

**Published:** 2020-06-26

**Authors:** Naphatsanan Duansak, Pritsana Piyabhan, Umarat Srisawat, Jarinyaporn Naowaboot, Nusiri Lerdvuthisopon, Geert Schmid-Schönbein

**Affiliations:** ^1^Division of Physiology, Department of Preclinical Science, Faculty of Medicine, Thammasat University, Pathum Thani 12120, Thailand; ^2^Division of Pharmacology, Department of Preclinical Science, Faculty of Medicine, Thammasat University, Pathum Thani 12120, Thailand; ^3^Division of Biochemistry, Department of Preclinical Science, Faculty of Medicine, Thammasat University, Pathum Thani 12120, Thailand; ^4^Department of Bioengineering, Institute of Engineering in Medicine, University of California, San Diego, La Jolla, CA, USA

## Abstract

**Background:**

Inflammation and hypertension are primary mechanisms involving in obesity-associated adverse effects of a high-fat diet. The aim of this study was to evaluate the effects of rice bran extract (RBE) on arterial blood pressure, hepatic steatosis, inflammation, and oxidative stress in high-fat diet (HFD)-induced obese mice.

**Methods:**

Male ICR mice were divided into four groups, including a normal-diet control group, a high-fat diet (HFD) (60% kcal from fat) group, an HFD group treated with RBE (220 mg/kg/day), and an HFD group treated with 1100 mg/kg/day for eight weeks. Besides body weight and arterial blood pressure, we determined liver values of total cholesterol, triglyceride, as well as percent body fat, tumor necrosis factor-*α* (TNF-*α*), malondialdehyde (MDA), nuclear factor kappa-B (NF-*κ*B), matrix metalloprotease-9 (MMP-9), cyclooxygenase-2 (COX-2), and mRNA endothelial nitric oxide synthase (eNOS).

**Results:**

The HFD group had increased body weight, increased systolic and diastolic blood pressure, liver total cholesterol, triglyceride, NF-*κ*B, COX-2 and MMP-9 protein levels, and decreased mRNA eNOS in the aorta. Mice of the HFD group receiving RBE had reduced diastolic blood pressure, as well as significantly decreased liver and serum TNF-*α* and MDA levels in the liver, and reduced NF-*κ*B levels in both the liver and heart.

**Conclusions:**

These results demonstrate that RBE decreases diastolic blood pressure, the liver lipid droplet accumulation, liver and myocardial NF-*κ*B, myocardial COX-2 and MMP-9 protein levels, and oxidative stress. Moreover, RBE may improve endothelial function and may alleviate adverse health effects associated with obesity including obesity-associated hypertension.

## 1. Introduction

Obesity as a global health problem is a major risk factor for metabolic diseases, such as hyperlipidemia, atherosclerosis, type 2 diabetes, fatty liver, and hypertension [1, 2]. Obesity and hypertension are also independent risk factors for the development of vascular dysfunction associated with low-grade chronic systemic inflammation, especially in adipose tissue. The inflammation is influenced by activation of the innate immune system in adipose tissue that promotes a proinflammatory status and oxidative stress, triggering a systemic acute-phase response. Several chronic diseases are the result of obesity associated with oxidative stress [3]. Excessive adipose tissue can serve as a source of enhanced levels of proinflammatory cytokines including tumor necrosis factor-alpha (TNF-*α*), interleukin (IL)-1*β*, and IL-6 [4]. TNF-*α* regulates the inflammatory response, adipose cell apoptosis, and lipid metabolism and peroxidation (malondialdehyde, MDA), increasing hepatic lipogenesis and insulin signaling and promoting NF-*κ*B signaling [5]. Furthermore, a high-fat and carbohydrate diet increases inflammation [6] and oxidative stress and reduces nitric oxide (NO) bioavailability to play a major role in endothelial dysfunction in obesity-associated hypertension [7, 8]. Rats fed HFD developed type 2 diabetes, insulin resistance, and liver inflammation with the presence of stenosis by increased hepatic TNF-*α*. In addition, these rats had increased cytochrome P4502E1 (CYP2E1) and oxidative stress with increased 4-hydroxynonenal [9]. Moreover, C57BL/6 mice with HFD become obese within weeks and, with prolonged feeding, they showed steatosis, decreased adiponectin, and increased serum glucose indicating insulin resistance hyperglycemia [10, 11].

The prevalence of obesity and associated complications, such as hypertension, has led to renewed research on traditional herbal medicines with negligible adverse effects, as alternative anti-inflammatory and weight loss therapies. Rice bran extract (RBE) is a source of *γ*-oryzanol, a mixture of lipids [12]. *γ*-Oryzanol has been associated with various beneficial effects, including anti-inflammatory and antihyperlipoproteinaemic properties, lowering of cholesterol values and platelet aggregation [13–18]. Earlier studies have reported that elevated blood pressure could be controlled with monounsaturated fatty acid and polyunsaturated fatty acid intake [19–21]. Since, RBE treatment in HFD showed the reduction of body weight, abdominal fat tissue weight, liver weight, and serum lipid levels [22]. However, mechanisms by which *γ*-oryzanol supplementation affects high-fat diet (HFD)-induced hypertension remain largely uncertain.

We hypothesized that RBE treatment during an HFD reduces obesity, hypertension, fat accumulation in the liver, and complications such as oxidative stress and inflammation in the heart. Accordingly, the aim of this study was to evaluate the effect of chronic oral RBE treatment on these pathological parameters in the liver, cardiac tissue, and aorta of mice consuming a high-caloric diet.

## 2. Methods

### 2.1. Animals and Experimental Model

All protocols were approved by the Animal Ethics Committee of Thammasat University (Pathum Thani, Thailand) for use and care of mice (rec. no. AE 018/2015). Male ICR mice (20–25 g) were obtained from the National Laboratory Animal Center of Mahidol University (Nakhon Pathom, Thailand). Mice were housed at 25 ± 2°C with a 12/12-light/dark cycle. Control mice were fed for 8 weeks on a control 70% kcal carbohydrate, 20% kcal protein, and 10% kcal fat (0.72 mg cholesterol by gram of lard), with a total energy of 3.85 kcal/g. An obese group of mice were fed, for 8 weeks, an HFD containing 20% kcal carbohydrate, 20% kcal protein, and 60% kcal fat (0.72 mg cholesterol by gram of lard), with a total energy of 5.24 kcal/g. Obese mice received the HFD with rice bran extract (RBE) (220 and 1100 mg/kg/day, respectively). All treatments were administered orally by feeding tubes every day for 8 weeks. At the end of the treatment, mice were fasted for 6 h and then given general anesthesia with 2% isoflurane by inhalation.

### 2.2. Chemicals and Reagents

All chemicals were purchased from Sigma-Aldrich (St. Louis, MO, USA). The HFD and normal diets (ND) were purchased from Research Diets (New Brunswick, NJ, USA).

### 2.3. Preparation and Characterization of Rice Bran Water Extract

The bran of Khao Dawk Mali 105 rice variety was purchased from the local mill in Surin Province, Thailand. Rice was grown in the organic farm approved by the Organic Agriculture Certification of the Department of Agricultural Extension (Bangkok, Thailand). Freshly milled rice bran was stabilized at 130°C for 90 sec. About 2,000 g of stabilized rice bran was boiled in 8,000 mL of distilled water for 1 h at 70°C. After centrifugation at 6,583 g for 10 min, the supernatant was freeze-dried (Lyophilization Systems Inc., USA) into a powdered extract. The procedure for preparation was previously described in detail [23]. The proximate analysis, total phenolic compounds, and *γ*-oryzanol contents of rice bran water extract were also determined using the method of the Association of Official Analytical Chemists, Folin–Ciocalteu method, and high-performance liquid chromatography [24], respectively. The yield of crude extract was 18%. The contents of total phenolic compounds were 4.6 mg gallic acid equivalents/g extract and *γ*-oryzanols were 4.6 *μ*g/g extract [22].

### 2.4. Tail-Cuff Blood Pressure

Mice were placed in a plastic tube restrainer. A pressure cuff with a pneumatic pulse sensor was attached to the tail. Mice were allowed to habituate to this procedure for 7 days before experiments were performed. Systolic and diastolic pressures were recorded after heating of the tail (model LE-5001, noninvasive blood pressure, Harlan, USA) and averaged over at least three consecutive readings per mouse [25, 26].

### 2.5. Cholesterol and Triglyceride in Liver Measurement

Liver total cholesterol (TC) and triglyceride (TG) levels were determined using a commercial kit (Wako, Osaka, Japan) as previously described [27] with some modifications. Briefly, liver (50 mg) was homogenized and extracted with 1 mL of isopropanol and centrifuged (8,000 rpm, 4°C) for 15 min, and the supernatant TG content was determined by using a commercial kit (Wako, Osaka, Japan).

### 2.6. TNF-*α* Measurement

Liver and serum were measured of TNF-*α* using mouse TNF-*α* ELISA (Affymetrix, eBioscience, USA) according to the instructions of the manufacturer.

### 2.7. Measurement of MDA in Liver

Liver MDA concentration was measured using a thiobarbituric acid reactive substance assay (Cayman Chemical, Ann Arbor, MI, USA). Livers were finely sliced and homogenized in 1.15% chilled potassium chloride. After 10 min of centrifugation (10,286 g, 4°C), the clear supernatant was collected. The liver MDA concentrations were immediately measured and normalized by the protein levels of homogenates. The protein values were determined using thiobarbituric acid reactivity (TBAR) based on the Bradford method [28].

### 2.8. Immunohistochemistry

Liver and heart samples were fixed in 10% formalin, embedded in paraffin, and sectioned for immunohistochemistry. To facilitate quantitative comparisons between groups, the sections were immunolabeled in a standardized procedure using anti-NF-*κ*B p65 rabbit polyclonal antibody (phospho S536 antibody, 100 *µ*l, Abcam, Cambridge, United Kingdom), anti-COX-2 rabbit monoclonal antibody (PM-9121-S1, Thermo Scientific), and anti-MMP-9 antibody (ab38898, Abcam) at 4°C overnight. The primary antibodies were visualized by binding of the secondary antibody conjugated to peroxidase activity with the diaminobenzidine (DAB) substrate (Vector NovaRED). Buffer alone or nonspecific purified rabbit immunoglobulin G (IgG) served as controls. After labeling with NF-*κ*B, digital light microscopy images of sections of the liver and the myocardium were captured (DAS-Fi2, Nikon, 10x eyepiece and 40x objective, digital camera, Olympus, Tokyo, Japan). NF-*κ*B, COX-2, and MMP-9 immunolabel density was measured by light intensity values (using ImageJ software with digital units between 0 = white and 255 = black) on at least 10 randomly selected windows (100 *µ*m × 100 *μ*m) per section. In each window, the digital light intensity was determined as the sum of the light intensities of all pixels divided by the number of pixels. Four random sections per organ were used to determine the mean optical densities in each animal. All measurements were carried out under standardized light microscopy settings.

### 2.9. Histological Analysis

A portion of liver tissues were fixed in 10% formalin, embedded in paraffin, sectioned, and stained with hematoxylin and eosin (H & E). Histological images were examined by digital light microscopy.

### 2.10. Real-Time Quantitative RT-PCR

The total RNA from the aorta was prepared using an extraction reagent (GenUP^™^ Total RNA Kit, Biotechrabbit GmbH, Berlin, Germany) according to the manufacturer's instructions. The RNA was reverse-transcribed using a high capacity reverse transcription kit (QuantiTect Reverse Transcription Kit, Qiagen, Hilden, German) according to the manufacturer's instructions. The real-time PCR was carried out using SYBR Green PCR Master Mix (QPCR Green Master Mix, Biotechrabbit) according to the manufacturer's instructions on a StepOnePlus Real-Time PCR System (Applied Biosystems, Foster City, USA). The PCR reaction consisted of 10 *μ*L of 2x SYBR Green PCR Master Mix, 1 *μ*L of forward and reverse primers (final concentration 0.5 *μ*M), 4 *μ*L of cDNA template (40 ng), and 5 *μ*L of nuclease-free water. The PCR conditions were a denaturing step at 95°C for 2 min, followed by 40 cycles at 95°C for 15 sec and 63°C–65°C for 30 sec. Melt curve analysis was performed at the end of PCR to access whether a single, specific PCR product was obtained. The specificity of PCR product was also analyzed by 1.5% (*w*/*v*) agarose gel electrophoresis with ethidium bromide. The molecular weight of the target DNA was identified by a 100-bp DNA ladder. The PCR data were normalized by the internal control beta-actin. The fold change of expression (the treated group vs. the control group) was calculated using a 2^−ΔΔCt^ method [29]. The 2^−ΔΔCt^ method to analyze RT-PCR data assumes that the real-time PCR assay has 100% amplification efficiency. The amplification efficiency of each primer set was quantified to indicate the accuracy and reliability of this analyzing method. The PCR primers used for gene expression analysis [30, 31] are shown in Table 1.

### 2.11. Statistical Analyses

Data represented are presented as mean ± SEM, *n* = 6 mice. Two-way analysis of variance (ANOVA) followed by Tukey's post hoc test was used to compare the results. A value of *P* < 0.05 was considered statistically significant.

## 3. Results

### 3.1. Food Intake, Body Weight, and White Adipose Tissue

The weight of mice fed the HFD after 8 weeks was higher than that of mice fed the normal control diet. The RBE treatment reduced body weight compared to the high-fat diet group (Table 2). The epididymal fat pad weight of the HFD group was increased as compared with the normal-diet group. However, the fat weight was significantly decreased in RBE-treated groups at all doses. The average daily food intake throughout the experimental period was also higher in the HFD than in the control group and remained elevated in the HFD group during RBE treatment (Table 2).

### 3.2. Blood Pressure

Systolic and diastolic blood pressures were markedly upregulated in HFD mice compared with control mice on normal diet (Table 3). The RBE treatment served to reduce the systolic and diastolic blood pressure in mice on HFD.

### 3.3. Effects of RBE on the Liver

**Figure 1:**
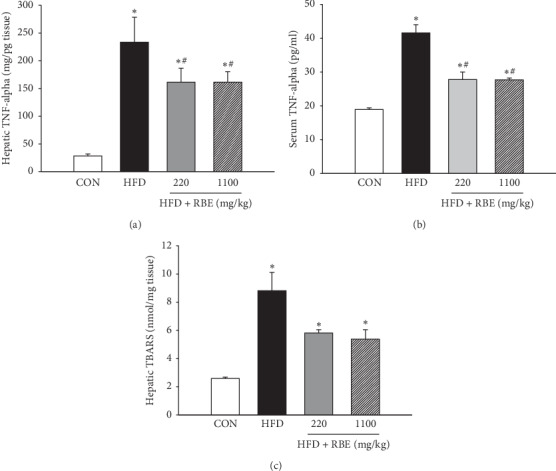
Effect of RBE on liver TNF-*α* (a), serum TNF-*α* (b), and liver TBARS (c). ^*∗*^*P* < 0.05 when compared to the normal control group; ^#^*P* < 0.05 when compared to the HFD group.

The hepatic and serum TNF-*α* level was increased in the HFD group as compare to the control group. Interestingly, the RBE treatment suppressed the TNF-*α* expression (Figures 1(a) and 1(b)) in control or high-fat diet. The high-fat diet group had increased liver TBARS compared with the normal control group, and the increase in TBARS was exacerbated further in HFD-RBE (Figure 1(c)). These findings suggest that high-fat diet increased oxidative stress and inflammation in obese mice.

**Figure 2:**
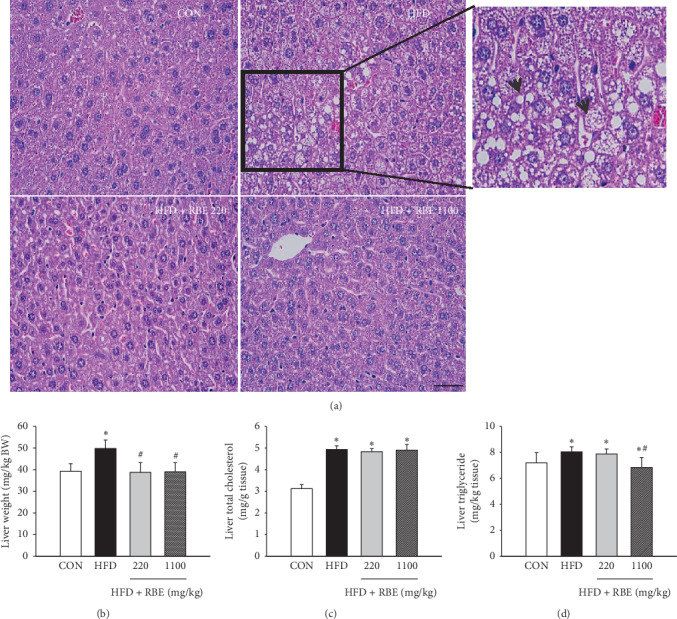
Effect of RBE administration for 8 weeks on lipid accumulation in the liver of HFD-induced obese mice. Liver tissue was stained with hematoxylin and eosin. Length bar = 50 *μ*m. (a) The RBE treatment decreased lipid accumulation in the liver. Hepatocytes of obese mice were filled with macrovesicular fat deposits while microvesicular fat deposits were found to a lesser extent in obese mice treated with RBE. Arrowheads indicate lipid droplets. Effect of RBE on liver weight (b), total cholesterol (c), and triglyceride (d) in high-fat diet-induced obese mice. ^*∗*^*P* < 0.05 when compared to the normal control group.

Histological analysis showed a marked accumulation of fat in the liver of the HFD group (Figure 2(a)) as compared to a low-fat accumulation in the liver of the HFD group treated with RBE. The triglyceride and total cholesterol levels of mice on high-fat diet were higher than those of mice on normal diet (Figures 2(b) and 2(c)). The average liver weight was decreased in the HFD-RBE-treated mice compared to HFD mice (Figure 2(d)).

### 3.4. NF-*κ*B and COX-2 Label Density after RBE Consumption

**Figure 3:**
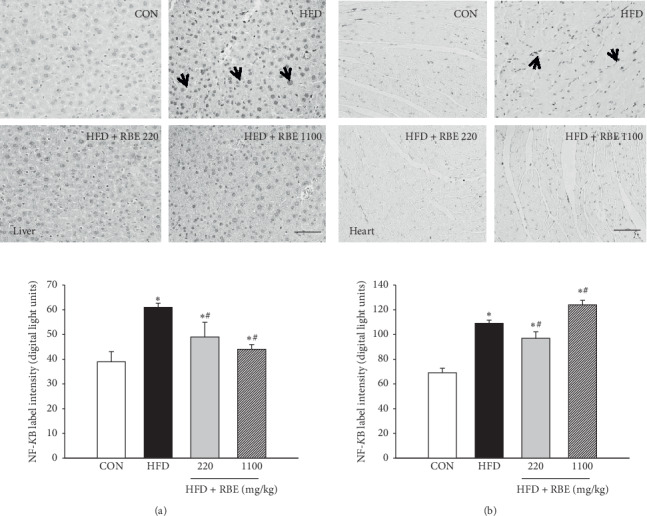
Effect of RBE on (a) hepatic NF-*κ*B and on (b) myocardium NF-*κ*B. ^*∗*^*P* < 0.05 compared to CON; ^#^*P* < 0.05 compared to the HFD group. Scale = 50 *μ*m.

**Figure 4:**
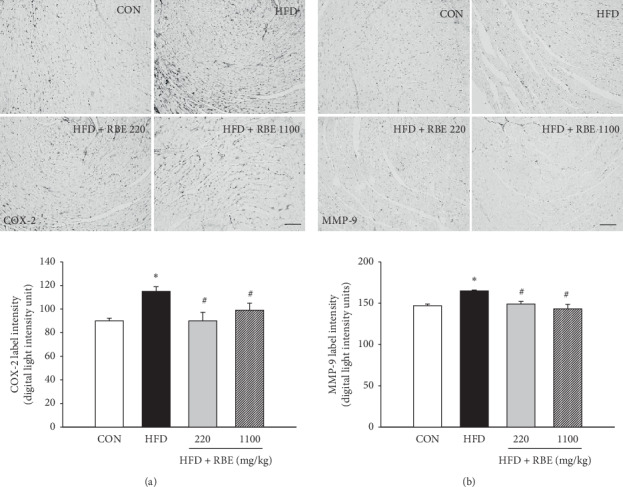
Immunohistochemical images of myocardial (a) COX-2 and (b) MMP-9 (scale bar = 100 *μ*m) with digital measurements (bar graphs). ^*∗*^*P* < 0.05 compared to CON; ^#^*P* < 0.05 compared to the HFD group.

The HFD group, compared with the normal-diet group, had an average of 50% higher NF-*κ*B immunolabel densities in hepatic and 57% in myocardial tissue (*P* < 0.05). The NF-*κ*B levels in the liver and myocardium were significantly decreased in the treatment groups (Figures 3(a) and 3(b)), whereas the HFD group has elevated COX-2 label density in the myocardium compared with the normal-diet group (Figure 4(a)), and the RBE treatment significantly decreased it.

### 3.5. Myocardium MMP-9 Expression

Extensive changes in ECM remodeling have also been shown in cardiac tissue using immunohistochemistry involving MMP-9. The MMP-9 level was reduced in RBE-treated mice on HFD compared with untreated mice fed HFD (Figure 4(b)).

### 3.6. Effects of RBE on Vascular eNOS mRNA Expression

**Figure 5:**
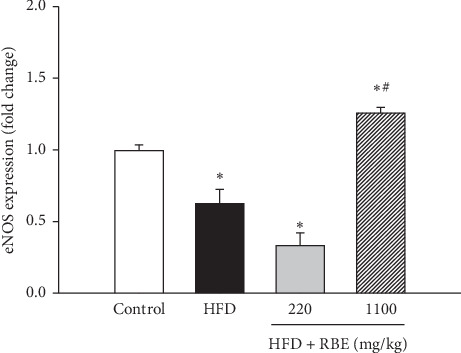
mRNA expression of eNOS in the aorta. Results are expressed as fold change over mice fed a control diet (CON). ^*∗*^*P* < 0.05 compared to the normal control group. ^#^*P* < 0.05 compared to the HFD group.

The expression level of vascular eNOS mRNA in the HFD group was reduced compared to that in the normal-diet group. However, its expression in the high-dose RBE-treated groups was upregulated compared to that of the HFD group (Figure 5).

## 4. Discussion

The current model of high-fat diet, extensively described in the past [32, 33], supports the notion that hypertension is associated with visceral adiposity [34]. Both obesity and hypertension exacerbate inﬂammatory markers and oxidative stress. Treatment with a traditional rice bran water extract markedly decreased the adiposity in high-fat-diet mice, their hypertensive state as well as their hepatic and myocardial inflammatory markers, and oxidative damages. This evidence points towards a broader range of actions by this traditional medicine.

Our results are in agreement with those of Kang et al. showing that treatment with rice bran causes a significant decrease in plasma and erythrocyte MDA levels in mice on high-fat diet [35]. Rice bran enzymatic extract reduces the production of superoxide anion in the aorta of obese rats [36] and also significantly inhibits high-fat diet-induced NF-kB p65 expression in mice. These results are in line with studies reporting decreased levels of proinflammatory markers (e.g., TNF-*α* and inducible nitric oxide synthase) in the vasculature and serum of obese Zucker rats after supplementation with the rice bran enzymatic extract [36]. Since oxidative stress and/or TNF-*α* activate intracellular transcription factor NF-*κ*B, which after translocation from the cytoplasm into the nucleus in turn activates downstream inﬂammatory cytokines [37–39], RBE may influence multiple inflammatory pathways. There is robust evidence for vascular endothelial dysfunction in hypertension. Obesity is often accompanied by a state of inflammation (vascular and systemic) that can cause endothelial dysfunction. Activation of NF-*κ*B [40], due to fat accumulation, serves to establish a proinflammatory and prothrombotic state, indicating the presence of altered vascular function that predisposes to the development of hypertension.

Inflammation and oxidative stress are associated with endothelial dysfunction and vascular hypertrophy, in which microvascular endothelium-dependent vasodilation is impaired in response to vasodilators such as insulin [41]. Insulin signaling pathways in vascular endothelial cell are based on the balance between vasoconstriction and vasodilatation. Free fatty acids, angiotensin II, MMPs, and other proinflammatory molecules induce hypertension and vascular insulin resistance. The question is whether such proinflammatory signals can be counteracted by RBE. *γ*-Oryzanol has direct effects on the glucose regulation. It reduces the risk of high-fat diet-induced hyperglycemia via regulation of insulin secretion as well as the activities of hepatic glucose-regulating enzymes such as G6Pase and phosphoenolpyruvate carboxykinase (PEPCK) [42]. *γ*-Oryzanol treatment stimulates GLUT4 translocation into the plasma membrane and glucose uptake, suggesting that this may be a mechanism by which *γ*-oryzanol regulates hyperglycemia [43]. Interestingly, the treatment with RBE resulted in decreased weight gain, serum insulin levels, expression of the lipogenic gene, and fat deposition in the pancreas, and increased cardiac free radical-scavenging activity in HFD rats [22, 44, 45]. In this study, the dose of RBE was 220 and 1100 mg/kg BW, which is equivalent to about 2.14 and 10.70 gm of daily intake by humans. This is a value that may be reached during daily RBE intake, depending on the level of intestinal absorption. However, concentrations in patient plasma remain to be determined.

## 5. Conclusion

In conclusion, rice bran extract administration has a broad effect on fat accumulation, oxidative stress, and blood pressure in HFD-induced obese mice, including (a) reduction of total cholesterol, triglyceride, and lipid accumulation in the liver, (b) reduction of the arterial blood pressure, and (c) attenuation of important inflammatory markers, such as NF-*κ*B, COX-2 and MMP-9 protein levels. The mechanisms required for RBE to reduce weight gain and food intake during HFD need further clarification, especially the role of leptin and its receptor. The effect of RBE on inflammatory mediators in obese patients should be determined.

## Figures and Tables

**Table 1:** Sequences of primers used for the RT-PCR analysis.

Genes	Primer sequences	Product length (bp)
eNOS3	Forward: 5′-TGTCACTATGGCAACCAGCGT-3′	148
	Reverse: 5′-GCGCAATGTGAGTCCGAAAA-3′	

GAPDH	Forward: 5′-ACCCCAGCAAGGACACTGAGCAAG-3′	92
	Reverse: 5′-TGGGGGTCTGGGATGGAAATTGTG-3′	

**Table 2:** Effect of RBE on food intake, body weight, and tissue weight.

	CON	HFD	HFD-RBE 220	HFD-RBE 1100
Food intake (g/day/mouse)	3.1 ± 0.1	3.6 ± 0.1	3.7 ± 0.2	3.8 ± 0.5
Final body weight (g)	37.5 ± 0.8	45.9 ± 0.8^*∗*^	40.5 ± 0.4^*∗*^	38.9 ± 0.6^*∗*^^#^
White adipose tissue weight (g)	0.29 ± 0.11	0.74 ± 0.11^*∗*^	0.53 ± 0.27^*∗*^	0.48 ± .023^#^

Values are mean ± SEM. ^*∗*^*P* < 0.05 when compared to the control; ^#^*P* < 0.05 when compared to the HFD group. CON: normal mice on control diet; HFD: HFD-induced obese mice; HFD + RBE: HFD-induced obese mice + RBE 220 and 1100 mg/kg (*n* = 6 rats/group).

**Table 3:** Effects of RBE on blood pressure.

	Control	HFD	HFD-RBE 220	HFD-RBE 1100
Systolic (mmHg)	127 ± 10	199 ± 20^*∗*^	182 ± 12^*∗*^	183 ± 11^*∗*^
Diastolic (mmHg)	85 ± 7	169 ± 15^*∗*^	131 ± 25^*∗*^^#^	116 ± 20^*∗*^^#^

Values are mean ± SEM. ^*∗*^*P* < 0.05 when compared to the normal; ^#^*P* < 0.05 when compared to the HFD group (*n* = 6 rats/group).

## Data Availability

The generated and analyzed data used to support the findings of this study are included within the article.

## Abbreviations

RBE: Rice bran extract
HFD: High-fat diet
TNF-*α*: Tumor necrosis factor-*α*
MDA: Malondialdehyde
NF-*κ*B: Nuclear factor kappa-B
MMP-9: Matrix metalloprotease-9
COX-2: Cyclooxygenase-2
eNOS: Endothelial nitric oxide synthase
TC: Total cholesterol
TG: Triglyceride
IL-1*β*: Interleukin-1*β*
NO: Nitric oxide
CYP2E1: Cytochrome P4502E1.
