# Corrigendum to “The Effect of Rice Bran Extract on Arterial Blood Pressure, Hepatic Steatosis, and Inflammation in Mice Fed with a High-Fat Diet”

**DOI:** 10.1155/2020/7461763

**Published:** 2020-12-14

**Authors:** Naphatsanan Duansak, Pritsana Piyabhan, Umarat Srisawat, Jarinyaporn Naowaboot, Nusiri Lerdvuthisopon, Geert Schmid-Schönbein

**Affiliations:** ^1^Division of Physiology, Department of Preclinical Science, Faculty of Medicine, Thammasat University, Klong Luang, Pathum Thani, Thailand; ^2^Division of Pharmacology, Department of Preclinical Science, Faculty of Medicine, Thammasat University, Klong Luang, Pathum Thani, Thailand; ^3^Division of Biochemistry, Department of Preclinical Science, Faculty of Medicine, Thammasat University, Klong Luang, Pathum Thani, Thailand; ^4^Department of Bioengineering, Institute of Engineering in Medicine, University of California, La Jolla, San Diego, CA, USA

In the article titled “The Effect of Rice Bran Extract on Arterial Blood Pressure, Hepatic Steatosis, and Inflammation in Mice Fed with a High-Fat Diet” [1], the “Conflicts of Interest” section should read as follows.

## References

[1] Duansak N., Piyabhan P., Srisawat U., Naowaboot J., Lerdvuthisopon N., Schmid-Schönbein G. (2020). The effect of rice bran extract on arterial blood pressure, hepatic steatosis, and inflammation in mice fed with a high-fat diet. *Journal of Nutrition and Metabolism*.

